# COVID-19-related Rhino-orbital-cerebral Mucormycosis

**DOI:** 10.5339/qmj.2022.47

**Published:** 2022-10-06

**Authors:** Raid M. Al-Ani, Khalid Mohsin Al Tameemi

**Affiliations:** ^1^College of Medicine, University of Anbar, Anbar, Iraq med.raed.alani2003@uoanbar.edu.iq; ^2^Department of Surgery/Otolaryngology, Imam Hussein Medical City, Karbala, Iraq

**Keywords:** COVID-19, Rhino-orbital-cerebral mucormycosis, Mucormycosis, COVID-19 complications

## Abstract

Background: There is an increasing number of COVID-19-related Rhino-orbital-cerebral mucormycosis (ROCM), especially from India.

Objectives: To evaluate the demographic, clinical, radiological, and outcome of the COVID-19-related ROCM cases in a single center.

Materials and Methods: The medical records of the patients with COVID-19-related ROCM were retrospectively reviewed. The study covered 22 months duration from March 2020 to December 2021 in Imam Hussain Medical City, Karbala city, Iraq.

Results: Of the 14 patients with COVID-19-related ROCM, there were 71.4% males with a male-to-female ratio of 2.5:1. The median age was 61 years (age range: 27-80 years). There were 42.9% of patients with a history of being a current smoker. All patients had a history of DM, and 57.1% of patients had a history of hypertension. All patients were without a history of the previous episode of COVID-19 or taking the vaccine. The median duration from the COVID-19 diagnosis to the diagnosis of ROCM was 19 days (duration range of 10-40 days). Most of the cases were of severe type (57.1%). All of the patients were taking corticosteroid and oxygen therapy. Nasal obstruction, nasal discharge, cheek swelling, and necrotic tissue were clinical features in all patients. The majority of the cases were on the left side (71.4%). Stage 3 was found in 42.9%. Amphotericin B was used for all patients and surgical debridement in 13 cases. Five patients have died (35.7%).

Conclusion: COVID-19-related ROCM is an aggressive disease associated with a high mortality rate of 35.7%. Early diagnosis and on-time initiation of treatment are recommended to get the best outcome.

## Introduction

In the era of the COVID-19 pandemic, otolaryngology is basically a relevant medical branch to deal with this disease. In the beginning, a nasopharyngeal sampling swab was used to detect SARS-CoV-2 by reverse transcriptase-polymerase chain reaction (RT-PCR). The World Health Organization declared anosmia as a specific feature of COVID-19. The development of long-standing symptoms like parosmia, sudden sensorineural deafness, and dysphonia are also features of this devastating disease.^1,2^ Moreover, SARS-CoV-2 was detected in the middle ear. The most aggressive otolaryngology feature is the appearance of COVID-19-related rhino-orbital-cerebral mucormycosis (ROCM).^3^

Although mucormycosis occurs on rare occasions, it is an aggressive fungal disease with a high rate of morbidity and mortality. It is caused by different species of the order *Mucorales*. It usually affects individuals with an impaired immune system^4^.

Despite COVID-19-related ROCM cases being reported from different parts of the world, the majority of them were from India^5^. Also, in the pre-pandemic COVID-19 era, the largest proportion of ROCM cases was reported from India^6^. In a large case series from India of probable or proven 388 mucormycosis cases, diabetes mellitus (DM) and previous history of trauma were the most common risk factors^4^.

The head and neck are the most common parts of the body involved in mucormycosis. Other parts like the lung, skin, and kidney are also affected. The ROCM is considered the commonest presentation form. There are various clinical features, which depend on the involved site, including unilateral nasal obstruction, foul-smelling nasal discharge, cheek swelling, hemifacial pain, headache, decreased visual acuity, orbital pain, loss of vision, etc.^7^ Unilateral ROCM involvement of the paranasal sinuses (59%), orbit (63%), and central nervous system (16%) is more common than bilateral involvement (paranasal sinuses 40%, orbit (8.6%), and central nervous system 5%)^8^.

ROCM could be occurred during the active phase or as a long-term sequel of the COVID-19.^3^ Even though many possible mechanisms explained the causal relationship between COVID-19 and ROCM such as reduced immunity by SARS-CoV-2,^9^ DM,^3^ steroid therapy,^10^ and thromboembolic phenomena,^11^ the real mechanism remains not clearly understood.

The diagnosis of mucormycosis is usually possible if there is a peculiar clinical feature like black discoloration of the nose or the palate.^12^ Microscopic examination of the nasal swab mounted with potassium hydroxide (KOH) to detect the Mucorales species is a preliminary test. Histopathological evaluation of the excised tissues is a confirmatory test because it can distinguish the presence of the fungus as a pathogen in the specimen from a culture contaminant as well as confirmation of the presence of a blood vessel invasion.^7,13^ Direct microscopy of KOH wet mounts can be applied to all materials sent to the lab. It is preferable to use fluorescent brighteners like Blankophor and Calcofluor White as well as KOH, which aids in the visualization of the characteristic of the fungal hyphae, a fluorescent microscope is required to accomplish the examination.^14^ Radiological examination through computerized tomography (CT) scan and/or magnetic resonance imaging (MRI) is of paramount importance in describing the stage of the disease. A multidisciplinary team is required to treat the patient. In general, antifungal therapy like liposomal amphotericin B, surgical debridement, and reversal of any immune-compromised conditions, if applicable, are the treatment modalities of this devastating disease.^7^ Early diagnosis and prompt treatment are of utmost importance to getting better outcomes.^3^

A prior study from Basrah city/Iraq reported 32 cases of RCOM over 5 years period (2011-2016).^15^ In the COVID-19 pandemic era, another study from Basrah city reported a case of ROCM in a 53-year-old patient with type 2 DM as a complication of COVID-19. Although RCOM is an aggressive disease, there is no previous study in Karbala city about this subject, neither during the pre-COVID-19 pandemic nor during the period of the pandemic.^16^ This means that this disease rarely occurs in Iraq. Owing to the above-mentioned reason as well as an unusual increment in the cases of ROCM during the course of the disease or post-COVID-19 in our hospital, this work was initiated with a retrospective study of the ROCM cases regarding the various aspects of this aggressive disease. Furthermore, there was a need to explore if there are any differences of the disease behavior and management strategies between our country and various nations across the globe. Hence, this work aimes to present the relevant findings of 14 ROCM cases of those who are or have been previously COVID-19 positive.

## Materials and Methods

This retrospective observational study was conducted at Imam Hussain Medical City, Karbala, Iraq for 22 months (March 2020 to December 2021). Patients with active or recent COVID-19-related ROCM of any age and from both sexes were enrolled in the study. The diagnosis of COVID-19 depends on a positive RT-PCR of the nasopharyngeal swab. Recent COVID-19 subjects means patients who recovered from the disease up to three months before the presentation and now they were RT-PCR negative for SARS-CoV-2. The duration of three months was a highly acceptable definition of post-acute COVID-19 syndrome.^17^ Patients with ROCM not associated with COVID-19 or those with incomplete data in their medical records were excluded from the study.

The study was approved by the Medical Research Bioethical Committee of the College of Medicine, University of Karbala (reference number 28, on 13-6-2022). Informed consent was waived owing to the retrospective nature of the study. However, informed consent was taken from some patients about the publication of their images here in this study.

Data were collected from the medical records of the patients regarding the age, gender, COVID-19 vaccine status, previous attack of COVID-19, duration and severity of the COVID-19, use of corticosteroid, oxygen, and mechanical ventilation, comorbidities, side, clinical features, radiological investigations, stage of the ROCM, histological diagnosis, treatment modality, and outcome.

The severity of the symptomatic COVID-19 patients was divided into four categories:^18^

• Mild (low-grade fever, mild cough, and slight fatigue)
• Moderate (high-grade fever and moderate respiratory symptoms. Findings of pneumonitis were seen in chest X-ray)
• Severe (dyspnea, respiratory rate 30/min, blood oxygen saturation 93%, partial pressure of arterial oxygen to fraction of inspired oxygen ratio 300 mm Hg, or CT scans showing at least a 50% increase in infiltrating volume over 48–24 hours)
• Critical (respiratory failure, septic shock, and/or multiple-organ dysfunction or failure)

The COVID-19-related ROCM was staged according to the proposed staging system adopted by Honavar.^19^ Only proved cases of ROCM were enrolled in the current study. ROCM was proved with the histological examination of the nasal tissue biopsy. All subjects were followed up for a minimum of 6 months after their discharge from the hospital.

The data were entered and analyzed using Statistical Package for the Social Sciences (SPSS) version 26. For continuous variables, the median and interquartile range were calculated. While the categorical variables were presented in simple tables as frequencies and percentages.

## Results

Of the 14 patients with COVID-19-related ROCM, there were 10 (71.4%) males with a male-to-female ratio of 2.5:1. The median age was 61 years with an IQR of 49.5-61 years. There were 6 (42.9%) patients with a history of a current smoker. All patients were with a history of DM and 8 (57.1%) patients with a history of hypertension (Table 1).

All patients were without a history of the previous episode of COVID-19 nor took a vaccine against the disease. The median duration from the COVID-19 diagnosis to the diagnosis of ROCM was 19 days with an IQR of 13.5-19 days. Most of the cases were of severe type (n = 8, 57.1%). All of the patients were taking corticosteroid and oxygen therapy during the hospitalization period as per the treatment protocol for COVID-19 (Table 2).

**Figure 1.**
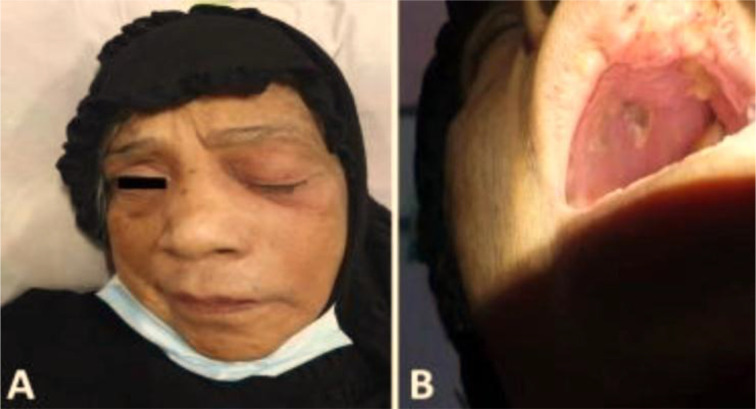
Black left-sided discoloration of the palate with proptosis in a 70-year-old female with COVID-19-related ROCM.

**Figure 2.**
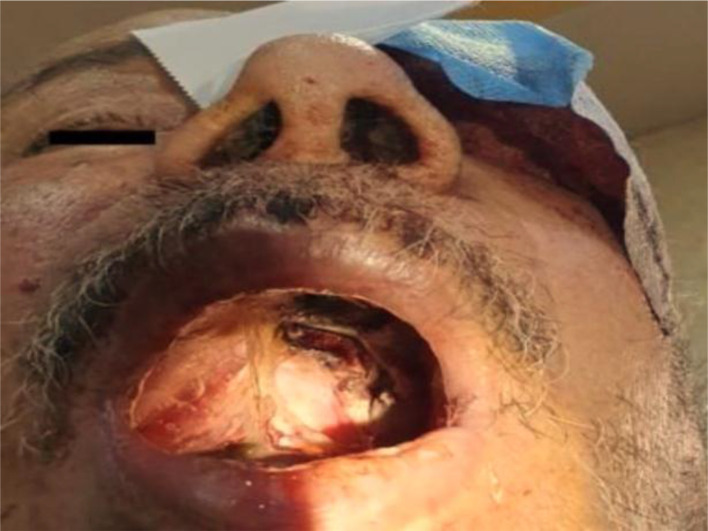
Black left-sided discoloration of the palate in a 66-year-old male with COVID-19-related ROCM.

**Figure 3.**
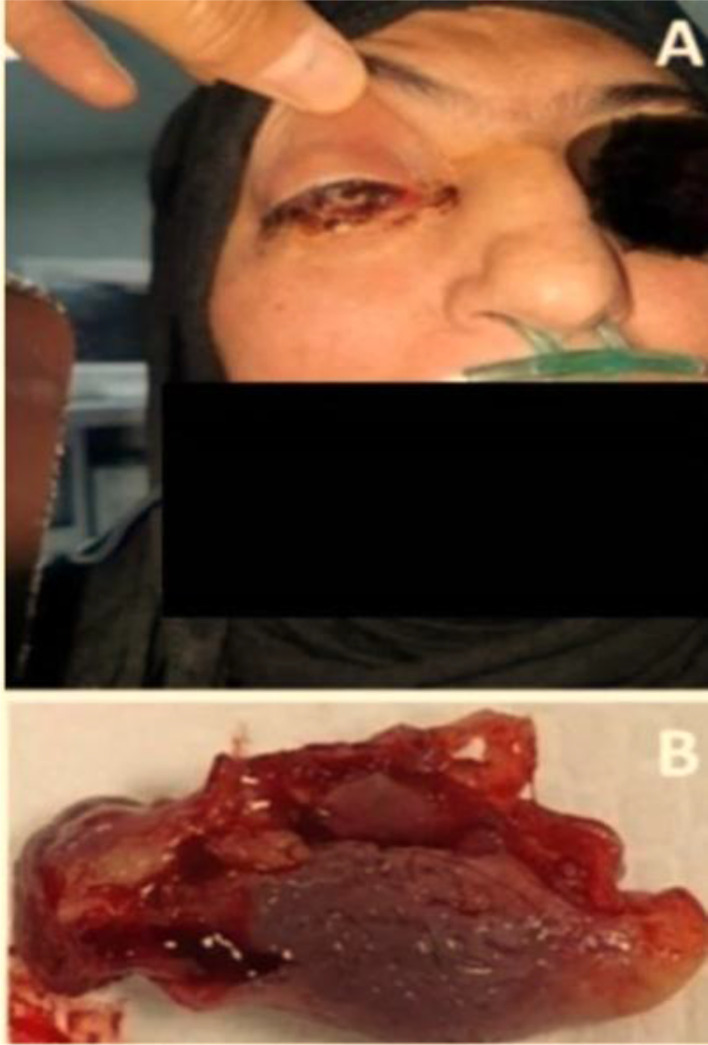
A 48-year-old female with stage 3 COVID-19-related ROCM (A). The excised right inferior turbinate (B).

**Figure 4.**
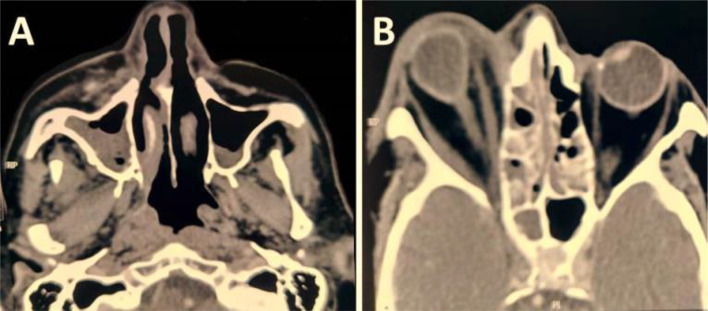
CT scan findings in a 68-year-old patient with COVID-19-related ROCM. A- Axial view of the nose and paranasal sinuses showing features of right maxillary sinusitis. B- Axial view showing right-sided proptosis, ethmoidal sinusitis, thickening of medial and lateral recti, and fat stranding in both intra- and extra-conal parts and the orbital apex.

Nasal obstruction, nasal discharge, cheek swelling, and necrotic tissue were clinical features in all patients, while epistaxis was the least feature of the disease (n = 1, 7.1%) (Table 3). Figures 1, 2, and 3 were examples of the features in some of our patients. All patients were diagnosed by histological examination of the nasal biopsies. CT scans were performed for all patients and MRI for those with orbital or central nervous tissue involvement, Figure 4 was an example of the findings of one of our patients with orbital involvement.

All patients with COVID-19-related ROCM were on one side with the majority of them on the left side (n = 10, 71.4%). Stage 3 was found in 6 cases (42.9%). Amphotericin B was used for all patients and surgical debridement in 13 (92.9%). Surgical debridement was not performed on one patient with a general condition (critical case of COVID-19) who was not allowed to perform the debridement. Five patients died (35.7%) (Table 4).

## Discussion

COVID-19 is a diagnostically problematic disease owing to the absence of classical presentation among the infected individuals. Although respiratory symptoms like cough and dyspnea are the most common presentation of the disease, other features such as smell and taste abnormalities, dysphonia, sudden sensorineural deafness, vertigo, dizziness, diarrhea, etc. might be seen in certain patients.^1,2^ Another feature of COVID-19 is the persistence of some features like dysphonia. It is well-known that this disease depresses the immune system of the affected subjects due to its ability to decrease the CD4 and CD8 cells as well as corticosteroid use as per the protocol of the disease management. As a consequence, there is a development of superadded or opportunistic infections in some patients. One of the more serious opportunistic fungal infections is mucormycosis. During the COVID-19 period, there is an unprecedented increase in mucormycosis cases, particularly rhino-orbital-cerebral type in various nations with the bulk of cases coming from India.^8^ Similar increment in the number of ROCM-related COVID-19 cases in comparison with a pre-pandemic period was also observed in this work. We reported that the disease is more prevalent in elderly and male patients. DM and corticosteroid therapy was the major predisposing factors. Early diagnosis and appropriate treatment of the disease had a better prognosis. The reported mortality rate in our case series study was 35.7%.

Mucormycosis is an aggressive opportunistic fungal infection caused by *Mucorales* species. Different parts of the body such as the nose, paranasal sinuses, eye, palate, jaws, central nervous system, pulmonary tissues, skin, kidney, and cardiac tissues are affected by the disease. Besides, a disseminated form can occur. However, ROCM constitutes around 2/3^rd^ of the cases.^20^ Following the inhalation of the fungal spores, angio-invasion, and necrosis of the nasal cavity begins and progresses into the neighboring structures; palate, paranasal sinuses, orbital cavity, and brain. In the pre-COVID-19 pandemic period, the global prevalence was 0.005 to 1.7/1,000,000 population. India is considered the most common endemic geographical area of the disease as it constitutes approximately 80 times more than other nations, 0.14/1000 population.^5,21,22^ Still, in the COVID-19 pandemic era, there is a large number of cases that came from India in comparison with a small number of case series or case reports from other parts of the world.^8,23^ The current study of a 14 case series is considered the first study from Iraq.

Many predisposing factors might lead to secondary infections, including factors related to the patients (like a history of DM, immunosuppressive drugs, and malignancy), hospital (nosocomial infection), and factors related to the COVID-19 (depress the immune system). One of the disaster infections is mucormycosis which carries a high rate of morbidity and mortality.^24^ As revealed by a prior study that 8% of hospitalized patients get secondary infections (bacterial or fungal). Another considerable factor is the widespread use of antibiotics without evidence of infection.^25^

Our study and previous studies reported that ROCM occurs during the active and recent stages of COVID-19.^8,26^ The median of the time elapsed between the diagnosis of COVID-19 and mucormycosis was 19 days (IQR = 10-40 days) in the current study which was much higher than the study by Pakdel et al., (median 7 days, range 1-37 days).^27^ Despite that this study did not find a cause for this difference, the dealing doctor needs to be aware of the occurrence of mucormycosis in the high-risk group of COVID-19 patients during the first 3 weeks. Furthermore, the current study results revealed that all patients did not have a previous attack of COVID-19 or take a vaccine against the disease. This means that the subsequent episodes after the first attack of the COVID-19 or those who were taking the vaccine have more powerful immunity and subsequently less severity of attacks if they are infected.

The current study reports that the median age of the patients was 61 years and there was a male predominance. These findings were consistent with previous studies.^7,26,28^ The possible cause of male preponderance may be attributed to that the immune response is stronger, which leads to more viral clearance in women than men as well as the antibody production is higher and lasts longer in women than men.^29^

The higher smoking rate (42.9%) among our patients in comparison with the general population might be explained that the ROCM-related COVID-19 is more prevalent in smokers, owing that smokers have already a negative impact on pulmonary function and lead to more severe stages of COVID-19.^30^

High blood sugar harms the function of the phagocytes as well as chemotaxis resulting in impairment of oxidative and non-oxidative mechanisms of the intracellular killing. All our patients had a recent or past history of DM and received corticosteroid therapy as part of the COVID-19 treatment regimen adopted by the Iraqi Ministry of Health. COVID-19 can harm the blood glucose level in patients with a history of DM. This effect might be due to the replication of the SARS-CoV-2 inside the human islet cells destroying β-cells. Besides, interleukin-6 (IL-6) can impair the phosphorylation of insulin receptors and insulin receptor substrate which leads to insulin resistance. Moreover, there are certain drugs such as corticosteroids, which are used in the management of patients with COVID-19, which can further increase blood glucose levels and more the patients for mucormycosis.

This study revealed that about 2/3^rd^ of the patients were at advanced stages (stages 3 and 4) at the time of the diagnosis. This finding was consistent with other previous investigations.^8,23,27^ This indicates that ROCM is an aggressive disease with rapid progression of the orbit and brain. Therefore, it is of utmost importance to catch the diagnosis as early as possible and this can be done by keeping the eye on those with risk factors like DM. Furthermore, it is advisable to give amphotericin B therapy as a prophylactic measure, particularly in immune-compromised patients, such as DM, or tapering the corticosteroids for a temporary period to control the infection.

The current recommendations consider that liposomal amphotericin B plus surgical debridement are the first options for the treatment of mucormycosis. While isavuconazole and posaconazole are considered the second option therapy.^31^ The mortality rate in our study was 35.7% despite all patients being treated with amphotericin B, and 13 patients were subjected to surgical debridement. As a result of the aggressiveness of the disease, early diagnosis and prompt antifungal treatment are highly recommended to reduce the fatality rate.^32^ A combination of antifungal therapy in a recent study achieved a 100% survival rate.^27^ The difference between the survival rate between the study of Pakdel et al.,^27^ and our study might be attributed to a combination of antifungal therapy being used in that study while amphotericin B alone was used in the current study. A delayed initiation with the antifungal drug is considered another factor. On August 5, 2022, the overall mortality rate due to COVID-19 in Iraq was 1.034 (25,321 deaths/2,448,484 confirmed cases) *https://covid19.who.int/region/emro/country/iq*. Owing to the large proportion of mucormycosis cases reported from India (80 times more than all the cases reported from the world^6^) the mortality rate from COVID-19- related ROCM in Iraq was 2.5 times than of what was reported from a large case series from India.^8^ Furthermore, the death rate due to COVID-19-related ROCM is approximately equal to the rate due to other causes.^4^ However, it is difficult to determine the exact mortality rate of the COVID-19-related ROCM because the death might be due to other complications of COVID-19 in certain patients. Therefore, a further autopsy study for patients with COVID-19-related ROCM to determine the exact cause of death is recommended.

The retrospective nature of this study was considered a limitation. Several issues were faced in collecting the data from the patients’ medical records because most of the subjects were on oxygen therapy or intubation. As such, taking a history from them as well as a physical examination for them was a little bit difficult. Therefore, we missed several cases of ROCM owing to the incomplete data in the patient's case sheets. However, the small sample size is an obstacle to the statistical analysis of the subgroups and was considered another shortcoming of the current study. The lack of a control group was a third limitation.

## Conclusion

The mortality rate in our case series was 35.7%. Most of the ROCM cases were seen during the first three weeks from the time of the diagnosis of COVID-19. Therefore, great care is necessary to catch the diagnosis as early as in these cases and treated them promptly to avoid the high rate of morbidity and mortality of this aggressive disease. Although the study gives an overview of this disease, its clinical significance remains limited. We recommend a future international study about the COVID-19-related ROCM to better understand the disease behavior and its management.

Conflict of Interest: The authors declare that there is no conflict of interest.

Funding: None

Ethics approval: The study was approved by the Medical Research Bioethical Committee of the College of Medicine, University of Karbala.

Consent to participate: Owing to the retrospective nature of the study, the consent from participants was waived

## Figures and Tables

**Table 1:** Characteristics of the 14 patients with COVID-19-related ROCM.

Variable	Frequency	Percent

Age (years)	Range = 27-80Median = 61IQR = 49.5-61	

Gender		

Male	10	71.4

Female	4	28.6

Smoking		

Yes	6	42.9

No	8	57.1

History of diabetes mellitus		

Recent	6	42.9

Past	8	57.1

History of hypertension		

Yes	8	57.1

No	6	42.9


**Table 2:** Characteristics of COVID-19 in 14 patients.

Variable	Frequency	Percent

Duration (days)	Range = 10-40Median = 19IQR = 13.5-19	

No previous COVID-19 attack	14	100

No vaccine	14	100

Severity		

Moderate	5	35.7

Severe	8	57.1

Critical	1	7.1

Steroid	14	100

Oxygen	14	100


**Table 3:** Clinical features of the COVID-19-related ROCM in 14 patients.

Variable	Frequency	Percent

Nasal obstruction	14	100

Offensive nasal discharge	14	100

Cheek swelling	14	100

Necrotic nasal tissue	14	100

Facial pain	12	85.7

Reduced visual acuity	11	78.6

Headache	8	57.1

Paraesthesia	7	50

Diplopia	7	50

Ophthalmoplegia	6	42.9

Loss of vision	5	35.7

Anesthesia	4	28.6

Proptosis	4	28.6

Altered sensorium	3	21.4

Necrotic palate	2	14.3

Epistaxis	1	7.1


**Table 4:** Characteristics of the COVID-19-related ROCM in 14 patients.

Variable	Frequency	Percent

Side		

Right	4	28.6

Left	10	71.4

Stage		

Stage 1	0	0

Stage 2	5	35.7

Stage 3	6	42.9

Stage 4	3	21.4

Amphotericin B	14	100

Debridement		

Yes	13	92.9

No	1	7.1

Outcome		

Live	9	64.3

Deceased	5	35.7

